# Attitudes of physicians from 10 European countries on adherence and how treatment modalities in ABSSSI affect adherence: results from a Delphi survey

**DOI:** 10.1007/s10096-018-3264-0

**Published:** 2018-06-06

**Authors:** Tom Stargardt, Christian Eckmann, Emilio Bouza, Gian Maria Rossolini, Paolo Antonio Grossi

**Affiliations:** 1 Hamburg Center for Health Economics, University of Hamburg, Esplanade 36, 20354 Hamburg, Germany; 2 Department of General, Visceral, and Thoracic Surgery, Klinikum Peine, Academic Hospital of Medical University Hannover, Hannover, Germany; 3 Department of Clinical Microbiology and Infectious Diseases, Hospital General Universitario Gregorio Marañón, Madrid, Spain; 4 Instituto de Investigación Sanitaria Gregorio Marañón, Madrid, Spain; 5 Medicine Department, School of Medicine, Universidad Complutense de Madrid, Madrid, Spain; 6 CIBER Enfermedades Respiratorias-CIBERES (CB06/06/0058), Madrid, Spain; 7 Department of Experimental and Clinical Medicine, University of Florence, Florence, Italy; 8 Microbiology and Virology Unit, Florence Careggi University Hospital, Florence, Italy; 9 Department of Medicine and Surgery, Section of Infectious Diseases, University of Insubria, Varese, Italy

**Keywords:** Adherence, Long-acting agents, Skin/soft-tissue infections, Skin structure infections, Intravenous route, Oral switch

## Abstract

To explore the attitudes of European physicians on adherence and how treatment modalities impact adherence in complicated forms of soft skin and skin structure infections, now referred as acute bacterial skin and skin structures infections (ABSSSI). After literature review, a questionnaire was prepared. Topics focused on (1) the importance of adherence, (2) the importance of administration regimen on adherence, (3) the importance of drug selection on adherence, (4) the importance of complexity on choice of drug for treatment, (5) the role of adherence in drug resistance, and (6) the role of adherence in administration of long-acting antibiotics (ABs). The questionnaire was administered to 323 European infectious diseases specialists, of whom 74% responded. A modified Delphi method was used to obtain the highest consensus. Results varied by countries. We found a high degree of agreement of the importance of adherence in ABSSSI treatment. Experts agreed that complexity of patient’s conditions, drug selection, drug resistance, the type of regimen, and the number of infusions impact adherence. Two items linking oral switching and adherence did not reach consensus. Adherence for ABSSSI therapies appears a crucial factor for therapeutic management and reduces the risk of AB resistance. Among new treatment opportunities, long-acting agents, with their characteristics, may represent an interesting options.

**Electronic supplementary material:**

The online version of this article (10.1007/s10096-018-3264-0) contains supplementary material, which is available to authorized users.

## Introduction

Acute bacterial skin and skin structure infections (ABSSSI) are one of the most frequent causes of hospitalization among skin and soft-tissue infections (SSTIs) [1, 2]. Management of ABSSSI is complex and includes surgical procedures and antibiotic (AB) treatment [1, 3].

Adherence to medication is strictly associated with treatments’ efficacy [4–6]. It is not considered a major issue for hospitalized ABSSSI patients, usually treated via intravenous (IV) route, but it may impact on outpatient treatments. High frequency of comorbidities, dose adjusting, and considerations for drug interactions raise complexity in drug selection and patient’s management [7]. Current treatments for ABSSSI often require multiple daily administrations, although in some cases, once-a-day dosing may be applicable [8]. Since these medications are often associated with gastrointestinal adverse events, a low adherence, and poor clinical outcome can occur [6]. Furthermore, AB misuse, and length of treatment might lead to drug resistance [5, 9]. Guidelines focusing on the issue of resistance frequently do not take into account variation in epidemiology of resistant pathogens, across countries [10].

Long-acting agents, which are characterized by a more favorable administration regimen, have been launched in the European markets [1, 11]. Their indications cover the major causes of ABSSSI, including MRSA. Among long-acting agents, dalbavicin has demonstrated an efficacy and safety comparable to other similar agents in the treatment of ABSSSIs, both in hospital wards and outpatient’s settings [12]. Its long half-life might be considered definitive advantages for the therapeutic treatment of ABSSSI patients and in terms of hospital cost-effectiveness [12].

The importance of adherence, for patients with ABSSSI, has not been addressed in therapeutic management guidelines, yet [7, 9, 13]. It is thus the aim of the present paper to explore the attitudes of European physicians on adherence and how treatment modalities impact on adherence.

## Methods

### Delphi method

A modified Delphi method was performed, aimed to reach the best estimate of consensus on unanswered questions [14]. As usual, each expert freely, and anonymously, delivers his/her opinion; an administrator provides a summary of the experts’ answers and their rationale. Typically, the process ends when an agreement has been reached on all the discussed topics, through multiple rounds of discussions. Here, we decided not to perform a second round, in favor of highlighting differences across countries.

### Delphi questionnaire preparation and administration

We performed a literature review on the importance of adherence in the management of ABSSSI and the impact of long-acting agents on adherence. In a first meeting, the authors of this paper identified the topics to address and drafted the Delphi questionnaire. Four external validators pre-tested the questionnaire, and as a result, the following topics were prepared:

1. Importance of the adherence for the treatment of ABSSSI
2. Importance of administration regimen for adherence in the treatment of ABSSSI
3. Impact of drug selection for the treatment of ABSSSI on adherence
4. Importance of complexity on choice of drug for the treatment of ABSSSI
5. Impact of drug resistance on the treatment of ABSSSI
6. Long-acting antibiotic (AB) and adherence

Each statement included three or more items.

Thereafter, the questionnaire was administered, via online software, to 323 European experts, from the following countries: Greece, Spain, Portugal, Russia, Bulgaria, Czech Republic, Austria, Romania, Poland, and Italy. The experts were chosen based on their practical experience in infectious diseases and were invited to share comments on the topic.

Each expert expressed his/her level of agreement according to the following 5-point Likert scale: 1 = strongly disagree, 2 = disagree, 3 = agree, 4 = more than agree, and 5 = strongly agree. An item achieved consensus, when the sum of items 1 and 2 (negative consensus) or 3, 4, and 5 (positive consensus) was ≥ 66%. No consensus was reached, when the sum of the responses for a negative consensus or a positive consensus was < 66%. Items 1 and 3, listed in topic 6, were purposely provided as internal controls, to evaluate the whole questionnaire reliability.

## Results

### Information on the participants

Two hundred thirty-eight among 323 (74%) experts from ten European countries participated in this survey. Most of the participants were infectious diseases specialists, while the remaining were clinical microbiologists, anesthesiologists, orthopedics, surgeons, or specialists in hygiene, pharmacology, dermatology, cardiovascular diseases, and other specialties (Table 1).

**Table 1:** List of the medical specialties participating at the survey

Specialties	%
Infectious disease	61%
Microbiology	8%
Anesthesiology/ICU	8%
Orthopedics	6%
Surgery/general surgery	5%
Hygiene	2%
Pharmacology	2%
Dermatology	1%
Cardiovascular	1%
Other field/department	8%
Total	100%

Table 2 lists the number of participants from each country with the following being the most numerous: Italy, Spain, Greece, and Romania.

**Table 2:** Distribution of the respondents, across the participating countries

Country	Total	Respondents	% of respondents
Austria	20	9	4%
Bulgaria	7	5	2%
Czech Rep	30	21	9%
Greece	34	31	13%
Italy	82	58	24%
Poland	31	14	6%
Portugal	28	18	8%
Romania	28	28	12%
Russia	4	1	0%
Spain	59	53	22%
Total	*323*	*238*	*100%*

Number in italics indicates the total

### Delphi survey: general results

The results of the survey are listed in Table 3. The first topic focused on the “Importance of adherence for the treatment of ABSSSI” (Table 3, topic 1). According to the participants, adherence to treatment exerts an important role in drug selection (item 4, 93% agreed) and can influence the outcome of treatment (item 2, 99% agreed) and the chance of relapse and recurrence of ABSSSI (item 5, 96% agreed). Moreover, it is a frequent issue encountered by the responding clinicians (item 1, 96% agreed) in their daily practice.

**Table 3:** Topics addressed in our survey

Topics	Level of agreement				
Importance of the adherence for the treatment of ABSSSI					
Items					
*1. Adherence is very important in the treatment of ABSSSI in your daily clinical practice*	4	6	47	84	98
	*4%*		*96%*		
*2. Adherence can influence the clinical outcome of therapy*	1	0	28	77	132
	*1%*		*99%*		
*3. Lack of adherence is a major problem in the treatment of ABSSSI in your daily clinical practice*	9	54	86	58	31
	26%		74%		
*4. The adherence has a major impact on drug selection*	0	17	86	86	48
	7%		93%		
*5. Lack of adherence has an impact on relapse and recurrence of ABSSSI and increases resources use*	0	10	58	102	68
	4%		96%		
Importance of administration regimen for adherence in the treatment of ABSSSI					
Items					
*1. Multiple infusions are a problem for patients in terms of adherence*	3	24	94	68	50
	11%		89%		
*2. Multiple infusions are a problem for the healthcare professionals (HCP) in managing patient adherence*	3	28	75	89	43
	13%		87%		
*3. Duration of infusion is a problem in managing patient adherence*	7	43	88	72	28
	21%		79%		
*4. I’ll be confident in using a long-acting antibiotic in patients with severe renal impairment if the drug can be removed by hemodialysis or adjusted in its dose*	4	21	95	82	35
	10%		90%		
*5. Oral-switch option after discharge could lead to a decrease of adherence*	10	86	86	37	18
	40%		60%		
*6. Poor adherence due to oral-switch can delay the discharge*	6	67	86	60	20
	31%		69%		
*7. Easier administration regimen reduces resources use*	1	6	68	89	74
	3%		97%		
Impact of drug selection for the treatment of ABSSSI on adherence					
*Items*					
*1. The need for dose adjustment and TDM increases complexity of patient management and thus reduces adherence*	7	24	71	91	45
	13%		87%		
*2. The need for dose adjustment is a major issue for adherence in patients with comorbidities and polypharmacy*	0	24	71	91	52
	10%		90%		
*3. The need for dose adjustment is a major issue for adherence in elderly patients*	0	35	60	98	45
	15%		85%		
*4. Multiple dose administration, dose adjustment and TDM increase healthcare utilization*	0	9	60	96	74
	3%		97%		
Importance of complexity on choice of drug for the treatment of ABSSSI					
*Items*					
*1. Level of complexity of treatment is important for the choice of drug*	1	9	79	88	61
	4%		96%		
*2. The need for a peripheral or central venous catheter for a prolonged period can increase complexity of treatment*	1	3	30	89	115
	2%		98%		
*3. The prolonged stay of intravenous devices for drug administration increase the risk for super-infections*	1	3	14	62	157
	2%		98%		
*4. Higher complexity increases time the HCP spend for the patient*	1	7	43	109	78
	3%		97%		
*5. Drug-drug interaction increase the complexity in management of patients*	1	0	47	109	81
	0%		100%		
*6. Super-infections increase the length of stay*	0	3	10	54	171
	1%		99%		
Impact of drug resistance on the treatment of ABSSSI					
*Items*					
*1. Resistance has an impact on relapse and on the recurrence of ABSSSI*	0	9	48	77	105
	3%		97%		
*2. ABSSSI due to resistant pathogen increases use of resources*	0	3	31	79	125
	1%		99%		
*3. ABSSSI due to resistant strain (e.g., MRSA) increase complexity of treatment*	0	4	38	89	106
	2%		98%		
*4. MRSA is a frequent cause of ABSSSI in my clinical practice*	9	64	69	71	26
	30%		70%		
*5. Under-exposure to treatment increases the risk of resistance selection*	0	7	44	91	96
	3%		97%		
*6. Under-exposure can be consequent to lack of adherence*	0	11	61	92	74
	5%		95%		
Long-acting AB and adherence					
*Items*					
*1. One single infusion to treat ABSSSI improves adherence*	1	0	33	65	139
	0%		100%		
*2. Switching to oral treatment for several days improves adherence*	11	92	61	55	18
	43%		57%		
*3. Multiple daily infusions improve adherence*	54	157	14	10	3
	89%		11%		

*ABSSSI* acute bacterial skin and skin structure infections, *AB* antibiotic, *HCP* healthcare professionals, *MRSA* methicillin-resistant *Staphylococcus aureus*, *TDM* therapeutic drug monitoring

The second topic regarded the “importance of administration regimen for adherence in the treatment of ABSSSI” (Table 3, topic 2). The number (item 1, 89% agreed) and the length of infusions (item 3, 79% agreed) were considered important elements for adherence to treatment. In order to keep patients adherent, the number of infusions (item 2, 87% agreed) and their administration by healthcare professionals (HCP) (item 7, 97% agreed) were also considered a burden. Oral switch may have a negative impact on adherence (item 6, 69% agreed). Long-acting ABs for ABSSSI patients were considered useful in patients with severe renal impairment, if drug hemodialysis or dose adjustment are accessible (item 4, 90% agreed). Item 5, the link between the existence of an oral switch option and adherence, did not achieve consensus (40% disagreed/60% agreed).

Topic 3 focused on “the impact of drug selection for the treatment of ABSSSI on adherence” (Table 3). Dose adjustment and therapeutic drug monitoring (TDM) increase therapeutic management and affect adherence, according to the experts (item 1, 87% agreed) especially in cases of comorbidities and polypharmacy (item 2, 90% agreed) and for the elderly (item 3, 85% agreement). Multiple doses, dose adjustment, and TDM were considered factors burdening the healthcare system (item 4, 97% agreed).

Topic 4 was on “importance of complexity on choice of drug for the treatment of ABSSSI” (Table 3). The experts considered the complexity of the treatment regimen, an important element in the choice of drug (item 1, 96% agreed). Moreover, drug-drug interaction (item 5, 100% agreed) and the length of use of a catheter (item 2, 98%) were valued affecting the complexity of the treatment. Complicated situations were found to increase the HCP time spent for the patient (item 4, 97% agreed). Finally, a prolonged use of IV devices was judged crucial for the risk of super-infections (item 3, 98% agreed), and the main reason for an increase of length of hospitalization (item 6, 99% agreed).

According to the experts, resistance to AB triggers relapse and recurrence of ABSSSI (Table 3, topic 5, item 1, 97% agreed), and increases the use of HCP resources (item 2, 99% agreed). Underexposure to treatment was considered a result from lack of adherence (item 6, 95% agreed) and a source for drug resistance (item 5, 97% agreed). Unsurprisingly, resistant strains, such as MRSA, are frequently encountered in the expert’s clinical practice (item 4, 70% agreed) and were valued to increase complexity of ABSSSI treatment (item 3, 98% agreed).

Last topic was on “long-acting AB and adherence” (Table 3, topic 6). One single infusion was considered to improve adherence (item 1, 100% agreed), as opposite to multiple infusions (item 3, 89% disagreed). No agreement was achieved on item 2, the link between switching to oral treatment and adherence (40% disagreed/57% agreed).

### European diversity: description of the conflicting items

Among 31 proposed, the two items on oral switch-options and their link to adherence remained with no consensus (topic 2, item 5; topic 6, item 2). Therefore, we decided to analyze the conflicting results by country (Supplementary Information, S.I., Table 1). Results for Austria (9 respondents), Bulgaria (5), and Russia (1) have to be interpreted cautiously because of the low number of respondents.

For “Oral-switch” option after discharge which could lead to a decrease of adherence, experts in Austria, Greece, and Poland achieved a positive consensus (S.I., Table 1, topic 2, item 5, 78, 74, and 79% agreed, respectively); no consensus was obtained for Bulgaria, Czech Republic, Italy, Spain, and Romania; and a negative consensus was reached from Russia and Portugal respondents (S.I. Tables 1, 100 and 89% disagreed, respectively).

For topic 6, item 2, “switching to oral treatment for several days improves adherence”, Bulgaria, Czech Republic, Portugal, Romania, and Russia reached a positive consensus (S.I., Table 1, topic 6, item 2, 80, 71, 72, 72, and 100% agreed, respectively); Italy, Greece, and Poland did not reach consensus; and Austrian respondents reached a negative consensus (S.I., 78% disagreed).

## Discussion

Adherence to treatment represents a key factor for treatment efficacy, especially with antimicrobial drugs [5, 15]. Moreover, it has a more powerful impact in cases of prolonged, chronic, or acute infections, such as ABSSSI [15].

The aim of our survey was to explore the attitude of a panel of infectious experts across ten European countries, on adherence and how treatment modalities impact adherence for ABSSSI treatments. A six-topic questionnaire and the Delphi method were used to obtain the most accurate expert’s opinion and consensus.

Interestingly, 61% of the participants were infectious diseases specialist (Table 1). It is important for us to highlight recent findings demonstrating a crucial role for these specialists in the management of severe infections disease, including ABSSSI. Indeed, it appears that the use of suitable guidelines and infectious disease specialist represent an efficacious approach aimed at reducing the incidence of inappropriate therapies and increasing good outcome rates [16].

The participants fully agreed on the importance of adherence in ABSSSI treatment. Undeniably, poor adherence to treatments is a major determinant for therapeutic failure, because it can lead to lack of response, recurrence, and predisposes to AB resistance [9].

Administration regimen and drug selection for ABSSSI’s treatment emerged as crucial factors for adherence. After initial treatment, patients may be switched to a suitable oral AB, or to outpatient parenteral antibiotic therapy (OPAT) [2]. In general, outpatient costs are lower, but treatments often require multiple and long infusions, loading on the healthcare systems and HCP utilization [17]. However, OPAT has reached interesting success in several European countries, reducing the risks of hospital-related infections [6, 18]. Oral MRSA-active drugs are included in guidelines for treatment of ABSSSI, in order to achieve an early switch (ES) and favoring early discharge (ED) [1]. Recent trials have also supported the concomitant use of oral antimicrobial therapies with incision and drainage, in less severe cases of ABSSSI [19, 20].

Interestingly, the link between oral treatment and adherence after discharge (topic 2, item 5) and for several days (topic 6, item 2) has produced conflicting answers, in our survey. A further analysis of the responses revealed that for topic 2-item 5, Portugal (and the single respondent from Russia) provided a negative consensus. A positive consensus was obtained for this item from Austria, Poland, and Greece. A no consensus was obtained for Italy, Czech Republic, Bulgaria, Spain, and Romania.

The limited availability/accessibility of some oral ABs, especially for the MRSA strains, could explain the responses obtained. Indeed, linezolid is the only available oral option in Romania (www.anm.ro/anmdm/en/), while in Bulgaria are ampicillin, levofloxacin, and clindamycin (www.bda.bg/en/). In Spain and Italy, both linezolid and tedizolid are available; however, they may be provided only in hospitals, and for a limited period outside hospital (www.aemps.gob.es/en/, www.aifa.gov.it). In Czech Republic, oral available ABs are penicillin, clindamycin, cephalosporins (ceftaroline, cefuroxime, ceftazidime), and linezolid; tedizolid is not marketed, yet (www.cepha.cz). Physician and patient expectations should be added as variables to an oral switch decision [1, 21, 22].

Regarding topic 6-item 2 statement, we found that respondents from Bulgaria, Czech Republic, Portugal, Romania, and Russia provided a positive consensus; Austria provided a negative consensus, and no consensus was reached by Greece, Italy, Poland, and Spain. Indeed, elderly patients or those with expected low adherence outside of hospital might have negatively influenced clinician’s responses for this statement [22]. Furthermore, improved conditions might negatively influence adherence in these classes of patients. In addition, and in accordance with our findings, Eckmann et al. reported a low rate of oral switching in Greece, Italy, and Poland and Spain (2, 4.7, and 4.7%, respectively), emerging a disagreeing scenario in ABSSSI management in these countries [21]. Since, long-acting agents dalbavancin and oritavancin, and tedizolid have been proved to be statistically non-inferior to linezolid or to vancomycin, followed by oral linezolid [1, 23–25], they might possess the potential for ED and ES to oral regimens [1].

Drug selection requires evaluation of patient’s condition, dose adjusting, consideration of comorbidities, drug-drug interactions, and adherence [1, 5, 10]. In our survey, the participants agreed on the importance of drug selection on adherence, especially for elderly and in the presence of comorbidities.

In our survey, clinicians strongly agreed on complexity on choice of drug in particular ABSSSI cases, since length of treatment, hospital stays, an increased risk of IV-related infections, and onset of super-infections may occur [9]. These circumstances represent a major difficulty for ES and consequently for an ED [22, 26].

Adherence emerged as an important factor also for drug resistance. MRSA strains account for 16.7% of all S aureus isolate, being > 25% from Spain, Greece, Italy, and Portugal and > 50% in Romania (http://ecdc.europa.eu/en/healthtopics/antimicrobialresistance/database/Pages/map_reports.aspx). Treatment for SSTI/ABSSSI is established; however, no epidemiological data for resistance patterns and geographical regions are specified [10]. Stewardship programs for ES and ED are being implemented in European hospitals. Nevertheless, clinician misconceptions, practical considerations, organizational factors, and lack of awareness of IV to oral switch guidance might limit their operation [22].

The participants agreed on the positive impact of long-acting ABs on adherence. Oritavancin, dalbavancin, and tedizolid phosphate could represent an additional opportunity for ED of ABSSSI patients [9]. Vancomycin is a standard choice for ABSSSI treatments, including MRSA infections [13]. However, it needs frequent drug monitoring and is associated with risk of nephrotoxicity. Moreover, new resistant strains have emerged in the recent years, limiting its use [27]. Oritavancin, dalbavancin, and tedizolid offer more feasible treatments [8, 9, 28, 29], and no evidences for resistant strains have been reported, yet [28, 29]. In a recent retrospective analysis, long-acting agents demonstrated a reduction in patient discomfort and risks associated with frequent manipulation and also favored ED and the use of OPAT facilities [18]. Thus, a strong impact in terms of cost effectiveness for the use of hospital resources, in favor to OPAT facilities might occur [30].

This survey has limitations. First, questions related to oral treatment might have been subject to the responder’s personal interpretation, given to the multiple variables to take into consideration for oral switching, including healthcare systems policies, availability/accessibility of the oral AB, type of patient, and economic factors. Second, given the nature of experts anonymously giving their opinion, we lack information on them, such as the hospitals or the units where the responders work.

## Conclusions

Delphi panels are valuable tools to develop indicators for various diseases [14]. All participants have shown a high level of agreement on the topics proposed, indicating the high quality and strength of the questionnaire. The number and variety of participating specialists strongly empowered our survey. Moreover, it offered interesting elements of discussion on health care settings and treatment modalities, across the participating countries.

No studies have been performed addressing adherence for ABSSSI patients, either for standard or novel treatments. Our survey, for the first time, has addressed this issue, finding a general agreement, across 10 European countries specialists, on the importance of adherence in drug selection, regimens and emerging resistance in ABSSSI. Further studies are needed to evaluate their impact on adherence regarding specific ABSSSI treatments, hospital burdens, and IV-related infections, in order to find treatment options contributing to improve patient’s adherence on medication.

## Electronic supplementary material

ESM 1 (XLSX 243 kb)

## Abbreviations

MRSA: Methicillin-resistant *Staphylococcus aureus*
SSTI: Skin and soft-tissue infections
ABSSSI: Acute bacterial skin and skin structure infections
IV: Intravenous
ED: Early discharge
ES: Early switch
AB: Antibiotic
